# Cognitive dysfunction associated with anti-glutamic acid decarboxylase autoimmunity: a case-control study

**DOI:** 10.1186/1471-2377-13-76

**Published:** 2013-07-09

**Authors:** Masahito Takagi, Yasushi Ishigaki, Kenji Uno, Shojiro Sawada, Junta Imai, Keizo Kaneko, Yutaka Hasegawa, Tetsuya Yamada, Ai Tokita, Kazumi Iseki, Shigenori Kanno, Yoshiyuki Nishio, Hideki Katagiri, Etsuro Mori

**Affiliations:** 1 Department of Behavioral Neurology and Cognitive Neuroscience, Tohoku University Graduate School of Medicine, Sendai, Japan; 2 Department of Diabetes and Metabolism, Tohoku University Hospital, Sendai, Japan; 3 Division of Advanced Therapeutics for Metabolic Diseases, Center for Translational and Advanced Animal Research, Tohoku University Graduate School of Medicine, Sendai, Japan

**Keywords:** Anti-glutamic acid decarboxylase antibodies, Diabetes mellitus, Stiff-person syndrome, Cognitive impairment, GABAergic neural system, Voxel-based morphometry

## Abstract

**Background:**

Glutamic acid decarboxylase (GAD) is the rate-limiting enzyme in the synthesis of γ-aminobutyric acid (GABA). Anti-GAD antibodies (GADA) are associated with the progression of stiff person syndrome and other neurological diseases, as well as the immune-mediated (type 1) diabetes. GABA is one of the most widely distributed neurotransmitters, but the non-motor symptoms of GADA-positive patients are not well understood. Diabetes is increasingly recognized as a risk factor for dementia; however, the relationship between diabetes and dementia is controversial.

The objective of this study was to assess cognitive function in patients with GADA-positive diabetes using subjects with GADA-negative type 2 diabetes as controls.

**Methods:**

Twenty-one patients with GADA-positive diabetes (mean age 52.5 ± 12.3 years, mean duration 7.7 ± 6.6 years) and 19 control subjects with GADA-negative type 2 diabetes (mean age 53.4 ± 8.9 years, mean duration 12.5 ± 6.7) were included in the study. The subjects underwent extensive neuropsychological testing and brain MRI.

**Results:**

The neuropsychological test scores were lower in the GADA-positive group than the control group (GADA-negative). Twelve subjects (57%) in the GADA group and 4 subjects (21%) in the control group had low performances (p = 0.027). No statistically significant differences were found between the GADA and control groups regarding demographics, diabetic severity cardiovascular risks, cerebral T2 hyperintensities, white matter volume and gray matter volume.

**Conclusions:**

Our study showed that GADA-positive diabetic patients have an increased risk of cognitive decline compared to patients with type 2 diabetes of comparable diabetic severity. It also showed that GADA may be associated with isolated cognitive decline in the absence of other neurological complications.

## Background

Glutamic acid decarboxylase (GAD) is the rate-limiting enzyme in the synthesis of γ-aminobutyric acid (GABA), a major inhibitory neurotransmitter that modulates and synchronizes neural network activity in the CNS [1]. Two isoforms of the enzyme, GAD65 and GAD67, are found in the CNS. Autoantibodies (GADA) against GAD65 inhibit the activity of GAD65 and therefore impair GABA synthesis. Various neurological manifestations are associated with GADA, including stiff person syndrome (SPS), cerebellar ataxia, epilepsy, and limbic encephalitis [2]. Intrathecal GADA synthesis induces symptoms in the CNS, presumably by affecting the GABAergic system [3], which is involved in cognitive function. However, little is known regarding the relationship between GADA and cognitive impairment.

Serum GADA play a role in the pathogenesis of the immune-mediated (type 1) diabetes [4-6]. Immune-mediated diabetes accounts for 5-10% of patients with diabetes [7], and GADA was found in 92% of adult autoimmune diabetic patients [8]. Diabetic patients are at risk for dementia [9-11], especially vascular dementia, and Alzheimer’s disease [12,13]. We recently encountered a patient with GADA-positive diabetes who presented with language problems, short-term memory disturbance, and frontal dysfunction without any other neurological deficits, suggesting that GADA may cause dementia [14]. This observation led to the hypothesis that GADA cause cognitive deficits in patients with immune-mediated diabetes. To determine whether GADA are an independent risk factor for dementia, we assessed cognitive function in patients with GADA-positive diabetes and GADA-negative type 2 diabetes who were matched for age, education, and glycemic control.

## Methods

### Participants

We assessed 23 patients with GADA who presented at the outpatient clinic of our hospital between July 2010 and August 2011. Patients were selected according to the following criteria: adult-onset (after age 20) GADA-positive diabetes; no previous history of neurological disorders, including stroke, dementia, or severe psychiatric disorders; no contraindications for MRI; sufficient ability to take neuropsychological tests; no use of psychotropic medications; and an MRI free of major-artery stroke, strategic-infarct, and space-occupying lesions. Two patients were excluded because they had been diagnosed with GADA-associated neurological disorders (one the patient with cerebellar ataxia (GADA titer = 11300 U/ml) and one the patient with limbic encephalitis (GADA titer = 15200 U/ml)). Thus, 21 patients with GADA were included in this study (denoted the GADA group).

As a control group, we assessed individuals with type 2 GADA-negative diabetes. We stratified the GADA-positive subjects according to age, dividing into 3 age-groups (35-49, 50-64, and greater than 65 years). Control subjects corresponding to the age-range were chosen and were paired with a GADA-positive subject based on the severity of their hyperglycemia, which is represented by HbA1c values. Inclusion and exclusion criteria were the same as described for the GADA group.

The study was approved by the Medical Ethics Committee of Tohoku University Graduate School of Medicine and was conducted in accordance with the ethical standards established in the Declaration of Helsinki. Prior to the beginning of the study, written consent was obtained from each participant.

### Medical backgrounds

At the time of entry into the study, each subject underwent a structured, in-person interview of health and function, followed by the completion of a standard medical history form, physical and neurological examinations, and a series of neuropsychological tests. The subjects’ medical records were examined for their most recent medical conditions and comorbid conditions. In regards to diabetic microangiopathies, any stage of retinopathy was counted. We defined diabetic neuropathy as self-awareness of peripheral sensory or motor neuropathy after developing diabetes. Diabetic nephropathy was defined as microalbuminuria, persistent proteinuria, or increased serum creatinine that could not be explained by any other kidney disease. Information on hypertension, dyslipidemia, smoking habits, insulin use, severe hypoglycemic episodes [15], and HbA1c values were self-reported and were confirmed by their medical records. Years of education were self-reported. Serum GADA titers were measured using a commercially available kit (Cosmic Corporation, Tokyo, Japan), that provides a specific and sensitive method for evaluating GAD antibodies [16].

### Neurological examination and neuropsychological evaluation

Global cognitive function was assessed with the Mini-Mental State Examination (MMSE) [17]. Intellectual ability was measured using the Japanese version of the Wechsler Adult Intelligence scale, 3^rd^ edition (WAIS-III), which yields 3 IQ scores (full scale IQ (FIQ), performance IQ (PIQ), and verbal IQ (VIQ)) and 4 index scores (verbal comprehension (VC), perceptual organization (PO), working memory (WM), and processing speed (PS)). Memory function was assessed using the logical memory test (LMT), a sub-test that was derived from the Japanese version of the revised Wechsler Memory Scale (WMS-R), and the Rey Osterrieth Complex Figure Test (RCFT). Verbal fluency tests (VF) and the trail-making test (TMT) were used as measures of executive function. We also used the digit span test derived from the WAIS-III for evaluating sustained attention, and we administered a Stroop test to assess directed attention. We asked the housemates of the subjects to fill out Japanese versions of the Neuropsychiatric Inventory Questionnaire (NPI-Q) [18], a validated clinical instrument for evaluating the behavioral and psychological symptoms of dementia.

### MRI data acquisition and analysis

All of the patients underwent brain MRI (1.5-Tesla; Signa, General Electric Medical Systems, Milwaukee, WI) within a month of the neuropsychological evaluation. The MRI protocols included axial T2-weighted (repetition time, 4750 ms; echo time, 95.0 ms; matrix size, 320 × 224; field of view, 210 mm; slice thickness, 6 mm), fluid attenuated inversion-recovery sequence (repetition time, 11000 ms; echo time, 110.0 ms; inversion time, 2200 ms; matrix size, 256 × 192; field of view, 210 mm; slice thickness, 6 mm), and T1-weighted 3-dimensional spoiled gradient echo (3-D SPGR) scans (repetition time, 20 ms; echo time, 4.1 ms; number of acquisition, 1; flip angle, 30°; field of view, 250 mm; matrix size, 256 × 256; slice thickness, 1.5 mm). The MRI data were carefully inspected to detect ineligible participants and were evaluated by a trained neurologist (KI) who was blinded to patient history. Cerebral infarcts were defined as focal hyperintensities (3 mm in size or larger) on T2-weighted images with a corresponding prominent hypointensity on T1-weighted images [19]. The severity of the T2 signal hyperintensity lesions was assessed using the scale described by Scheltens et al. [20]. The semi-quantitative rating method was used to assess periventricular hyperintensities (PVH), white matter hyperintensities (WMH), basal ganglia hyperintensities (BGH), and infratentorial foci (ITF). The total score and sub-scores were used for analyses. To compare the gray matter volumes between the 2 groups, we performed voxel-based morphometry. The 3-D SPGR data were analyzed with SPM8 software (Wellcome Department of Cognitive Neurology, Institute of Neurology, London) running on Matlab 2008a (Math-Works, Natick, MA, USA). We used the VBM8 toolbox (http://dbm.neuro.uni-jena.de/vbm.html) with default parameters.

The images were bias-corrected, tissue classified, and registered using linear (12-parameter affine) and non-linear transformations [21]. Next, analyses were performed on the gray matter volume (GMV) and white matter volume (WMV), which were then multiplied by the non-linear components derived from the normalization matrix to locally preserve actual gray matter and white matter values respectively. Finally, the modulated volumes were smoothed with a Gaussian kernel of 8 mm full width at half maximum. Voxel-wise differences between the groups were examined using independent sample t-tests. To avoid possible edge effects between different tissue types, we excluded all voxels with gray matter values of less than 0.2 (absolute threshold masking). We applied a threshold of p < 0.001 with an extent of 5 voxels across the whole brain. Age, disease duration, and total intracranial volume (TIV) were used as covariates, meaning that all of the effects that could be explained by these parameters were removed from the data. TIV was computed from the 3 components of global tissue volume; GMV, WMV, and CSF, which were determined by counting the voxels in native dimensions. The volumes of GMV, WMV and TIV were used to calculate the ratio of GMV or WMV to TIV in each subject.

### Statistical analysis

The data were analyzed using SPSS for Windows (Version 19, IBM SPSS Statistics, Chicago, IL). The differences in the baseline data and neuropsychological test performance between the GADA and control groups were assessed with independent sample t-tests, Mann–Whitney U tests, or Fisher exact tests, as appropriate. Spearman rank correlations were calculated for the GADA titer and the results of cognitive tests. All of the statistical tests were 2-tailed, and the results were considered significant at a level of p < 0.05. No adjustment was made for multiple comparisons because of the exploratory nature of the study. The relative risk of low cognitive performance in the GADA group versus the control group was calculated according to the number of low performers in each group. WAIS-III sub-scales and memory-related tests (LMT and RCFT) were considered major outcome measures. Low performers were defined as those whose IQ scores or indices were less than 80, which represents the lower normal limit of each sub-scale in WAIS-III. Age-adjusted cut-off points were also used to define low performance on memory tests.

## Results

### Patient characteristics

The baseline characteristics of the subjects are shown in Table 1. Female subjects represented 66.7% of the subjects in the GADA group and 57.9% of the subjects in the control group. The 2 groups were well balanced for age, years of education, and HbA1c values. The diabetes disease duration, body weight, and body mass index were greater in the control group. The required insulin dose and insulin index (insulin doses per body weight) were not significantly different between the groups. The number of complications varied between the groups, but the difference was not significant (Table 2). Fewer patients in the GADA group had hypertension or dyslipidemia. The prevalence of diabetic microangiopathies was comparable between the groups. Severe hypoglycemic episodes were not reported in either group. GADA titers in the GADA group ranged from 3.3 to 14,000 U/ml (median = 30.15 U/ml).

**Table 1:** Demographic data

**Variables**	**GADA**	**Control**
	**(n = 21)**	**(n = 19)**
Mean Age, y (SD)	52.5 (12.3)	53.4 (8.9)
Sex ratio (F:M)	14:7	11:8
Education, y (SD)	13.1 (2.9)	13.1 (2.1)
Mean disease duration, y (SD)	7.7 (6.6)	12.5 (6.7) *
Mean HbA1c value, % (SD)	7.9 (1.6)	7.4 (1.3)
Mean daily insulin dose, units (SD)	36.2 (32.2)	23.5 (25.5)
Insulin index, units/kg/day (SD)	0.6 (0.4)	0.3 (0.3)
Weight, kg (SD)	60.8 (12.5)	72.5 (15.6) *
Height, m (SD)	1.62 (9.8)	1.62 (8.9)
BMI, kg/m^2^ (SD)	23.4 (5.5)	27.4 (4.9) *

Insulin index - units of insulin taken in a 24-h period divided by weight in kg.

Abbreviations: *GADA* glutamic acid decarboxylase antibody, *BMI* body mass index (weight in kg divided by height in m^2^).

* *p <* 0.05.

**Table 2:** Number (%) of cases with complications

**Variables**	**GADA**	**Control**
	**(n = 21)**	**(n = 19)**
Hypertension, n (%)	9 (43%)	12 (63%)
Dyslipidemia, n (%)	8 (38%)	10 (53%)
Current smoker, n (%)	2 (10%)	3 (15%)
Diabetic retinopathy, n (%)	3 (14%)	5 (26%)
Diabetic nephropathy, n (%)	4 (19%)	8 (42%)
Diabetic neuropathy, n (%)	4 (19%)	5 (26%)
Small vessel disease on MRI, n (%)	3 (14%)	5 (26%)

Fisher exact tests showed no significant differences.

Abbreviations: *GADA* glutamic acid decarboxylase antibody.

### Neurological examination

None of the subjects had subjective symptoms including oscillopsia. However, abnormal eye motion, characterized by a discordant horizontal pursuit and a difficulty in maintaining horizontal gaze, was detected in 2 (10%) out of 21 patients with GADA. A similar eye movement disorder was noted in the patient with GADA-associated cerebellar ataxia, who was excluded from the study, but was not seen in any of the patients in the control group.

### Neuropsychological assessment

Table 3 shows the results of the neuropsychological assessments. The WAIS-III showed significant differences in 3 sub-scales: the FIQ (GADA 94.0 ± 16.2, control 105.8 ± 11.8, p = 0.01), VIQ (GADA 96.0 ± 16.3, control 107.2 ± 13.9, p = 0.03), and PO (GADA 93.5 ± 14.4, control 102.5 ± 10.6, p = 0.03) scores were lower in the GADA group than in the control group. On the WAIS-III, 10 GADA-positive subjects and 4 control subjects were low performers, but the difference was insignificant (p = 0.105). All the subjects in both group performed better than the age-adjusted cut-off point on the LMT and on the RCFT tests, but 8 subjects only in the GADA group achieved scores below the cut-off point in the RCFT delayed-recall test (p = 0.004). The average scores for the RCFT delayed-recall test (GADA 18.5 ± 9.7, control 23.8 ± 4.8, p = 0.03) were significantly lower in the GADA group than in the control group. Collectively, 12 subjects (57%) in the GADA group and 4 subjects (21%) in the control group had low performances on at least one major outcome measure (p = 0.027). The relative risk for cognitive decline was 2.71 (95% CI = 1.05-6.99) in the GADA group. In addition, phonemic verbal fluency was lower in the GADA group than in the control group (GADA 24.6 ± 7.3, control 31.3 ± 8.2, p = 0.009). In the GADA group, the GADA titer was not significantly correlated with any of the neuropsychological assessments.

**Table 3:** Neuropsychological test scores and NPI-Q scores (mean (SD))

	**GADA**	**Control**	**p-values**
	**(n = 21)**	**(n = 19)**	
*Global cognitive functioning*			
MMSE	28.8 (1.7)	29.1 (1.4)	0.63
*Intellectual ability* (WAIS-III)			
FIQ	94.0 (16.2)	105.8 (11.8)	0.01
VIQ	96.0 (16.3)	107.2 (13.9)	0.03
PIQ	92.8 (16.2)	100.5 (12.1)	0.10
VC	97.6 (14.4)	106.0 (13.9)	0.07
PO	93.5 (14.4)	102.5 (10.6)	0.03
WM	93.6 (17.6)	99.7 (11.6)	0.20
PS	94.0 (16.0)	96.5 (13.7)	0.61
*Attention*			
Digit Span, forward	6.3 (1.3)	6.8 (1.2)	0.15
Digit Span, backward	5.0 (1.5)	4.8 (1.0)	0.78
Stroop Test	145.5 (82.0)	112.4 (39.3)	0.11
*Memory*			
LMT, immediate	23.8 (8.6)	26.3 (7.8)	0.34
LMT, delay	19.0 (7.7)	23.0 (7.5)	0.11
RCFT, copy	34.7 (2.7)	35.5 (1.2)	0.28
RCFT, immediate	19.1 (10.1)	23.8 (5.2)	0.07
RCFT, delay	18.5 (9.7)	23.8 (4.8)	0.03
RCFT, recognition	19.9 (2.1)	20.6 (2.1)	0.29
*Executive functions*			
VF, phonemic (Fu/A/Ni)	24.6 (7.3)	31.3 (8.2)	0.009
VF, category (Animal)	16.9 (4.1)	17.6 (4.2)	0.56
TMT, part A	49.4 (18.3)	45.1 (15.1)	0.43
TMT, part B	151.3 (122.0)	108.6 (66.2)	0.17
*Neuropsychiatric state*			
NPI-Q severity^†^	1.2 (2.0)	0.4 (0.9)	0.36
NPI-Q distress^†^	1.2 (2.5)	0.2 (0.5)	0.28

Higher scores indicate better performance on all of the neuropsychological tests except for the trail-making test and the Stroop test, in which higher scores indicate worse performance. High NPI-Q scores reflect worse neuropsychiatric status.

Abbreviations: *GADA* glutamic acid decarboxylase antibody, *LMT* logical memory test, *RCFT* Rey Osterrieth complex figure test, *VF* verbal fluency tests, *TMT* trail making test, *NPI-Q* neuropsychiatric inventory questionnaire.

† Mann-Whitney U test.

### Psychiatric assessment

The NPI-Q showed a neuropsychiatric feature of GADA group, but the differences between the 2 groups did not reach statistical significance. Six subjects (29%) in the GADA group had total severity scores ranging from 2 to 6 points, and 4 subjects (21%) in the control group scored from 1 to 3 points. Four neuropsychiatric symptom domains were positive only in the GADA group: delusions (n = 1), agitation/aggression (n = 2), euphoria/elation (n = 1), and apathy/indifference (n = 2). Five other domains were positive in small numbers of patients in both groups: dysphoria/depression (GADA, n = 3; control, n = 1), anxiety (GADA, n = 2; control, n = 2), disinhibition (GADA, n = 1; control, n = 1), irritability/lability (GADA, n = 4; control, n = 2), nighttime behavioral disturbances (GADA, n = 1; control, n = 1). The appetite/eating disturbances domain was positive in a patient in the control group. Hallucinations and aberrant motor behaviors were absent in both groups.

### Neuroimaging analysis

MRIs detected one or two small infarcts in the basal ganglia (n = 4), ventral posterolateral thalamic nuclei (n = 1), cerebellum (n = 1), or brain stem (n = 2) in 3 patients in the GADA group and 5 in the control group (p = 0.442), but these patients were asymptomatic. The total Scheltens scores were not significantly different (GADA 8.0 ± 8.9, control 4.3 ± 4.2). Voxel-based morphometry did not show any differences between the groups in either the regional or global gray matter volume (Table 4). The average ratio of gray matter to total intracranial volume was 0.42 ± 0.36 in the GADA group and 0.43 ± 0.34 in the control group.

**Table 4:** The results of the MRI studies (mean (SD))

	**GADA**	**Control**	**p-values**
	**(n = 21)**	**(n = 19)**	
Total Sheltens’ score^†^	8.0 (8.9)	4.3 (4.2)	0.61
PVH^†^	1.3 (2.2)	0.6 (1.2)	0.96
WMH^†^	3.3 (4.9)	1.0 (2.4)	0.24
BGH^†^	3.1 (2.5)	2.3 (2.1)	0.59
ITF^†^	0.2 (0.4)	0.4 (0.8)	0.85
VBM			
GMV	577 (59.9)	554 (66.6)	0.26
WMV	541.3 (80.5)	505.2 (46.0)	0.09
TIV	1365 (160.8)	1298 (104.1)	0.12
WM ratio (WM/TIV)	0.40 (0.02)	0.39 (0.02)	0.34
GM ratio (GM/TIV)	0.42 (0.04)	0.43 (0.03)	0.90

Abbreviations: *GADA* glutamic acid decarboxylase antibody, *PVH* periventricular hyperintensities, *WMH* white matter hyperintensities, *BGH* basal ganglia hyperintensities, *ITF* infratentorial foci, *VBM* voxel-based morphometry, *GMV* gray matter volume, *WMV* white matter volume, *TIV* total intracranial volume, *WM ratio* white matter ratio, *GM ratio* gray matter ratio.

† Mann-Whitney U test.

## Discussion

To our knowledge, this is the first study that describes the cognitive profile of patients with GADA-positive diabetes. The major outcomes suggest that patients with GADA-positive diabetes are at a higher risk of cognitive decline than patients with type 2 diabetes. The cognitive abilities of diabetic patients are influenced by multiple factors [22], including dyslipidemia [23], chronic hyperglycemia [24], recurrent severe hypoglycemic attack [25], and obesity and hypertension [26], which may enhance the risk of cerebral vascular disease or vascular dementia [27]. In our case-controlled study, patients with GADA-positive diabetes were compared to type 2 diabetics and were matched by age and glycemic control. The insulin dose, obesity, comorbidity, smoking, number of cerebrovascular risk factors, and incidence of diabetic microangiopathy in the control group were somewhat greater than in the GADA group, although white matter lesions, which are thought to be associated with cognitive impairment in diabetics [28], were comparable between the groups. Therefore, the patients in the control group are at a higher risk for vascular events and dementia.Nevertheless, the mean cognitive tests scores were consistently lower in the GADA group. We cannot attribute the cognitive decline detected in the present study to the severity of diabetes or vascular involvement because there were no differences in these factors were observed between the groups; therefore, we propose that cognitive impairment is associated with GADA.

The GADA-positive patients in the present study did not exhibit any motor symptoms. Cognitive decline was independent from other GADA-related neurological syndromes, including SPS and cerebellar ataxia. The only subtle neurological manifestation, other than cognitive impairment, was a disturbance in horizontal eye motion, which was noted in 2 patients in the GADA group. It was also noted in the patient with GADA-associated cerebellar ataxia, who was excluded from the present study, and our previously published case with GADA-positive diabetes and dementia [14]. Full range of motion and conjugate movement indicate preserved integrity of the neural pathway from the frontal eye field to the cranial nerves, but a difficulty in unilateral gaze-holding suggests a dysfunction of the cerebello-vestibular system [29]. A similar phenomenon was described in SPS, and disruption of GABAergic neurons was proposed as a pathogenic mechanism [30]. Therefore, subtle horizontal eye motion disturbance may be a specific (but not sensitive) sign suggesting the involvement of GADA in cognitive decline.

To date, only a few GADA-related disorders, such as stiff person plus syndrome and limbic encephalitis, have been associated with cognitive dysfunction. Memory disturbance is a principal symptom of these disorders, but the involvement of other cognitive domains has not been thoroughly investigated. The present study uncovered impairments in executive function, language, general intelligence, perceptual organization, and memory in GADA-positive patients.

The impairment of multiple cognitive domains is common in schizophrenic patients [31]. Recent advances have untangled the relationship between cognitive impairment in schizophrenic patients and the dysfunction of the GABAergic system. The GAD67 isoform plays a key role in schizophrenia-related cognitive impairment [32,33]. Decreased GABA levels in the prefrontal lobes are associated with the executive dysfunction and working memory dysfunction in schizophrenia [32,34]. In addition, hippocampal GABAergic interneurons function in memory formation by modulating pyramidal cell activity [35]. Thus, the interrupted GABAergic system affects the modulation of pyramidal cell activity in the cerebral cortex, which may result in cognitive dysfunction. Although no significant correlation was noted between serum GADA titer and cognitive function, the impairment of GABAergic neurotransmission seems to have a key role. The examination of the intrathecal GADA titer or other related antibodies might be relevant, which were not assessed in the present study. Despite an assumed breakdown in the cortical GABAergic neural system, it remains unclear if the cognitive tests detected specific cortical areas that were vulnerable to decline or global cerebral damage.

Voxel-based morphometry failed to show any differences in regional or total gray matter volume between the 2 groups, moreover, neither in white matter volume. This did not support the involvement of local or global cerebral atrophy associated with GADA and suggests that neurodegenerative pathologies and neuronal loss have little role in the cognitive impairment of GADA-positive patients. A randomized control study and several case reports suggest that GADA-associated neurological manifestations are reversible and are successfully treated by immunomodulating therapy [36,37]. If this is the case, the cognitive impairment can be resolved in a similar fashion. Further studies are needed to confirm this hypothesis.

The present study is not without limitations. The selection process used in clinic-based recruiting may bias the subject pool. In addition, previous neurological or psychiatric disease history might exclude meaningful patients from both groups. Because of the exploratory nature of this study, we did not perform any corrections for multiple comparisons. As the present study was cross-sectional, no information was available concerning the prognosis of cognitive decline, such as whether it was progressive or underwent spontaneous remission.Moreover, this study did not provide direct evidence for the CNS involvement of GADA, such as the intrathecal synthesis of GADA.

## Conclusions

In summary, we found that patients with GADA-positive diabetes are at a higher risk for cognitive decline than those with GADA-negative type 2 diabetes of comparable diabetic severity. GADA may mediate cognitive dysfunction by disrupting GABA production and may contribute to dementia in diabetics. Further studies, including cohort studies and longitudinal studies, are required to uncover the mechanism in more detail.

## Abbreviations

BGH: Basal ganglia hyperintensities; FIQ: Full scale IQ; GABA: γ-aminobutyric acid; GAD: Glutamic acid decarboxylase; GADA: Anti-glutamic acid decarboxylase antibodies; ITF: Infratentorial foci; GMV: Gray matter volume; LMT: The logical memory test; NPI-Q: The Neuropsychiatric Inventory Questionnaire; PIQ: Performance IQ; PO: Perceptual organization index; PS: Processing speed index; PVH: Periventricular hyperintensities; RCFT: The Rey Osterrieth Complex Figure Test; TIV: Total intracranial volume; TMT: The trail-making test; VC: Verbal comprehension index; VF: Verbal fluency tests; VIQ: Verbal IQ; WM: Working memory index; WMH: White matter hyperintensities; WMV: White matter volume; WMS-R: The revised Wechsler Memory Scale; WAIS-III: The Wechsler Adult Intelligence scale, 3^rd^ edition; 3-D SPGR: 3-dimensional spoiled gradient echo.

## Competing interests

The authors declare that they have no competing interests.

## Authors’ contributions

MT, YI, and EM conceived and designed this study. MT and EM drafted the manuscript. MT, YI, KU, SS, JI, KK, YH, TY and AT recruited the study participants and participated in the data collection. MT, KI, SK, YN and EM participated in the data analysis. All the authors read and approved the final manuscript.

## Pre-publication history

The pre-publication history for this paper can be accessed here:

http://www.biomedcentral.com/1471-2377/13/76/prepub
